# Direct evidence of milk consumption from ancient human dental calculus

**DOI:** 10.1038/srep07104

**Published:** 2014-11-27

**Authors:** C. Warinner, J. Hendy, C. Speller, E. Cappellini, R. Fischer, C. Trachsel, J. Arneborg, N. Lynnerup, O. E. Craig, D. M. Swallow, A. Fotakis, R. J. Christensen, J. V. Olsen, A. Liebert, N. Montalva, S. Fiddyment, S. Charlton, M. Mackie, A. Canci, A. Bouwman, F. Rühli, M. T. P. Gilbert, M. J. Collins

**Affiliations:** 1 Department of Anthropology, University of Oklahoma, Norman, OK, USA; 2 Institute of Evolutionary Medicine, University of Zürich, Zürich, Switzerland; 3 BioArCh, Department of Archaeology, University of York, York, UK; 4 Centre for GeoGenetics, Natural History Museum of Denmark, University of Copenhagen, Copenhagen, Denmark; 5 Henry Wellcome Building for Cellular and Molecular Physiology, Oxford, UK; 6 Functional Genomics Center Zürich, University of Zürich/Swiss Federal Institute of Technology (ETH) Zürich, Zürich, Switzerland; 7 National Museum of Denmark, Copenhagen, Denmark; 8 School of GeoSciences, University of Edinburgh, Edinburgh, United Kingdom; 9 Laboratory of Biological Anthropology, Institute of Forensic Medicine, Faculty of Health Sciences, University of Copenhagen, Denmark; 10 Research Department of Genetics, Evolution and Environment, University College London, London, UK; 11 Novo Nordisk Foundation Center for Protein Research, Faculty of Health and Medical Sciences, University of Copenhagen, Copenhagen, Denmark; 12 Department of Anthropology, University College London, London UK; 13 Dipartimento di Archeologia, Università degli Studi di Padova, Padova, Italy; 14 Trace and Environmental DNA Laboratory, Department of Environment and Agriculture, Curtin University, Perth, Australia

## Abstract

Milk is a major food of global economic importance, and its consumption is regarded as a classic example of gene-culture evolution. Humans have exploited animal milk as a food resource for at least 8500 years, but the origins, spread, and scale of dairying remain poorly understood. Indirect lines of evidence, such as lipid isotopic ratios of pottery residues, faunal mortality profiles, and lactase persistence allele frequencies, provide a partial picture of this process; however, in order to understand how, where, and when humans consumed milk products, it is necessary to link evidence of consumption directly to individuals and their dairy livestock. Here we report the first direct evidence of milk consumption, the whey protein β-lactoglobulin (BLG), preserved in human dental calculus from the Bronze Age (ca. 3000 BCE) to the present day. Using protein tandem mass spectrometry, we demonstrate that BLG is a species-specific biomarker of dairy consumption, and we identify individuals consuming cattle, sheep, and goat milk products in the archaeological record. We then apply this method to human dental calculus from Greenland's medieval Norse colonies, and report a decline of this biomarker leading up to the abandonment of the Norse Greenland colonies in the 15^th^ century CE.

Milk is a major nutritional resource. In addition to being a source of clean liquid (milk is 80–90% water), milk solids contain approximately 25–55% sugar (lactose), 25–45% fat, and 5–35% protein (caseins and whey proteins), as well as calcium, potassium, and B-vitamins^1^. Adoption of animal milk consumption by humans typically requires behavioral adaptations, such as culturing and curdling techniques, to remove or reduce the lactose content of milk in order to make dairy products digestible after infancy. Additionally, populations with long pastoralist traditions in Europe and India, East Africa, and the Arabian peninsula have also independently evolved lactase persistence (LP), a genetic adaptation in the regulation of the lactase gene (*LCT*) that allows continued adult digestion of milk^2^,^3^ (Fig. 1). LP is hailed as one of the clearest examples of gene-culture co-evolution in humans^4^, yet many fundamental aspects of its evolution remain unknown^5^,^6^,^7^,^8^,^9^ and the socioeconomic context and scale of prehistoric and historic dairying are only poorly understood.

The ability to directly identify milk consumption patterns in past populations would thus advance understanding of human dietary ecology, evolution, and cultural agency. However, previous attempts to directly measure milk consumption using bone calcium isotopes have proven unsuccessful^10^. Other milk biomarkers offer only indirect lines of evidence. For example, isotopic inference of milk lipids from pottery residues^11^ is the most widely used approach to identify dairying, but this method cannot discriminate species of origin, and reuse of communal vessels and exploitation of cervid adipose tissue^12^ both pose further challenges to interpretation. Milk proteins have also been recovered from food residues^13^,^14^,^15^, but such finds are exceptional and rare, and animal bone evidence for the exploitation of secondary products is limited by the availability of large, well preserved assemblages to provide interpretable mortality profiles^16^,^17^.

To address these problems we turned to dental calculus, a mineralized form of dental plaque that serves as a long-term reservoir of dietary biomolecules and microfossils^18^. Nearly ubiquitous in archaeological populations and sourced directly from the oral cavity, dental calculus presents a unique opportunity to access primary evidence of ancient diets at an individual level. In a recent study^18^, we identified the milk whey protein β-lactoglobulin (BLG) in the dental calculus of a modern Swiss dental patient using tandem mass spectrometry. This protein was also recently identified within preserved kefir cheese curds associated with the mummified remains of Bronze Age Xiaohe pastoralists (ca. 1980-1450 BC) in Xinjiang, China^15^. BLG is a lipocalin within the calycin superfamily of proteins^19^, and it is the dominant whey (milk serum) protein in ruminant milk, making up 11% of the total milk proteins and 50% of the whey proteins^20^. BLG offers many advantages as a milk biomarker^19^,^20^ including: 1) humans do not produce BLG and therefore the presence of the protein in dental calculus excludes a host origin; 2) BLG is present only in milk and thus it is a specific biomarker for this fluid; 3) BLG is more resistant to enzymatic degradation and microbial proteolysis than other milk proteins^21^; 4) BLG lacks close bacterial orthologs, making it readily identifiable against a background of bacterial proteins; 5) over half of the amino acid residues in BLG are variable among traditional dairy livestock, allowing genus and species discrimination between cattle, buffalo, sheep, goat, horse, donkey, and reindeer, among others (Supplementary Fig. S1); 6) BLG is the dominant protein in the whey fraction of milk and partitions with lactose during dairy processing, thereby making BLG a superior proxy for lactose than caseins or milk fats, which separate from the lactose-rich whey fraction during cheese and butter production^20^; 7) and finally, because BLG is identified directly from protein sequence data, uncertainties arising from indirect detection methods, such as isotopic analysis, are minimized.

This study seeks to employ shotgun protein analysis by liquid chromatography-tandem mass spectrometry to identify dairy consumption in the archaeological record. In this study, we analyzed 92 archaeological dental calculus samples selected from regions with (Europe and northern Southwest Asia) and without (Central West Africa) long-standing dairying traditions for the presence of BLG (Fig. 1; Table 1). After establishing that BLG can be detected in the dental calculus of archaeological populations from dairy-consuming regions, we then applied this approach to 6 additional medieval dental calculus specimens from the Norse Greenland sites Brattahlið (Qassiarsuk) and Sandnes (Kilaarsarfik). These sites date to a period during which a major dietary shift from ruminant dairy to marine resources has been previously hypothesized on the basis of bone stable isotope and zooarchaeological evidence^22^,^23^. We confirm that a decline in BLG observed at these sites is consistent with a dietary shift, and specifically diminished access to dairy products, leading up to the abandonment of the Norse Greenland colonies in the 15^th^ century CE.

## Results

### Lactase Persistence in Present Day Populations

**Figure 1:**
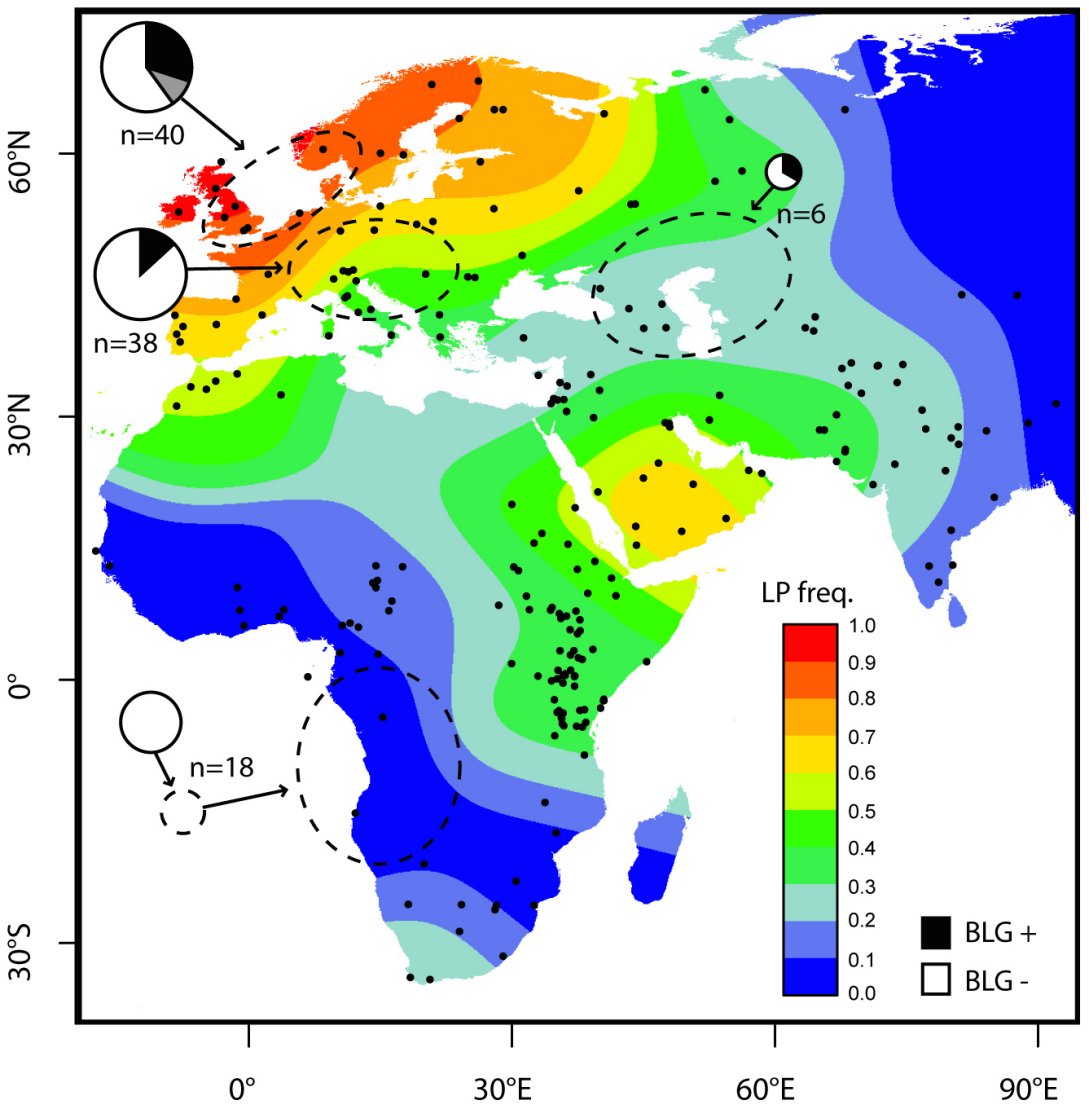
Locations of historic populations analyzed in this study and contour map of present day lactase persistence frequency inferred from LP frequency data. Archaeological dental calculus samples analyzed in this study were selected from regions (dashed ovals) where present day LP allele frequencies are high (Northern Europe: Britain, Norway, Denmark), moderate (Central Europe: Germany, Hungary, Italy), low (northern Southwest Asia: Armenia, Russia), and very low (Central West Africa, buried on the island of St. Helena). Pie charts for each region are scaled by sample size and indicate the proportion of individuals from each region testing positive for milk BLG peptides (black) in dental calculus. A pooled sample of five individuals from Norway testing positive for BLG is shown in gray indicating the uncertainty of the number of BLG+ individuals. Interpolated contour map of lactase persistence frequencies were generated from allele frequencies of all 5 known LP causal alleles (-13907*G, -13910*T, -13915*G, -14009*G and -14010*C) in present day populations in Europe, Africa, and northern Southwest Asia. The map was generated with the R *statspat* package^32^ using published data available as of March 2014 (see Methods section). Data points are shown as dots, and interpolation may be inaccurate where there are few data points.

To assist in the selection of archaeological samples for analysis, we generated an updated interpolated contour map of present day LP frequency in Europe, northern Southwest Asia, and Africa (Fig. 1) using recently published LP genotype data^24^,^25^. We then selected archaeological dental calculus specimens from sites located within regions with high (Northern Europe), moderate (Central Europe), low (northern Southwest Asia), and very low (Central West Africa) present-day LP frequencies.

### BLG in Europe and northern Southwest Asia

**Figure 2:**
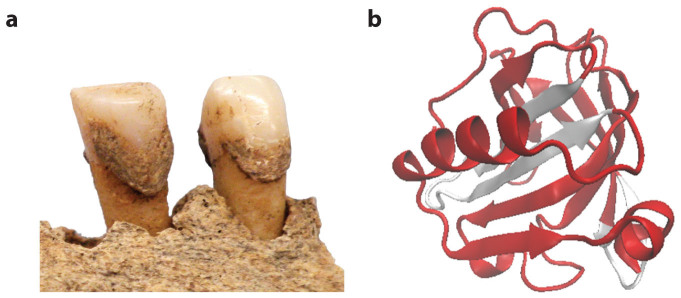
Protein coverage of β-lactoglobulin identified within Eurasian archaeological dental calculus. (a) Human dental calculus from the British Anglo-Saxon site of Norton-on-Tees (sample NEM18, ca. 6^th^ century CE) found to contain seven β-lactoglobulin peptides. (b) Three-dimensional structure of bovine β-lactoglobulin protein, rendered from PDB 3NPO using VMD v.1.9.1^36^. The mapped locations of all BLG peptide sequences identified by tandem mass spectrometry within archaeological dental calculus are shown in red, resulting in a coverage of 72% of the reconstructed consensus BLG protein.

Seventy-four dental calculus samples were selected from 20 sites in Northern Europe (Britain, Denmark, and Norway; n = 40), Central Europe (Germany, Hungary, and Italy; n = 38), and northern Southwest Asia (Armenia and Russia, n = 6) dating from the Bronze Age (ca. 3000 BCE) through the 19^th^ century CE (Table 1; Supplementary Table S1). Approximately one quarter (25.7%) of the Eurasian dental calculus samples tested positive for BLG peptides (Fig. 1). In total, 229 spectra (representing 37 unique peptide sequences) from the Eurasian dataset were assigned to BLG (Table 1; Supplementary Table S2), resulting in a reconstruction of 72% of the protein (Fig. 2). For each of the 19 Eurasian dental calculus samples that tested positive for BLG, the consensus BLG sequence could be assigned to ruminants of the Pecora infraorder of Artiodactyla, and 18 samples contained bovid-specific (Bovidae) peptides. Among these samples, 4 samples contained cattle-specific (*Bos* sp.) peptides, 3 samples contained sheep-specific (*Ovis* sp.) peptides, 2 samples contained goat-specific (*Capra* sp.) peptides, and 3 samples contained BLG peptides from multiple ruminant species (Supplementary Fig. S1; Supplementary Table S2).

### Absence of BLG in Central West Africa

Eighteen dental calculus samples were selected from a 19^th^ century cemetery on the island of St. Helena, located approximately 2,000 km west of Angola in the southern Atlantic Ocean. The cemetery contains the remains of Central West Africans^26^ originating from a region with traditionally very low or no milk consumption (Fig. 1; Supplementary Table S1). As expected, BLG peptides were not identified in any of the West African samples (Fig. 1; Table 1).

### BLG in Norse Greenland

**Figure 3:**
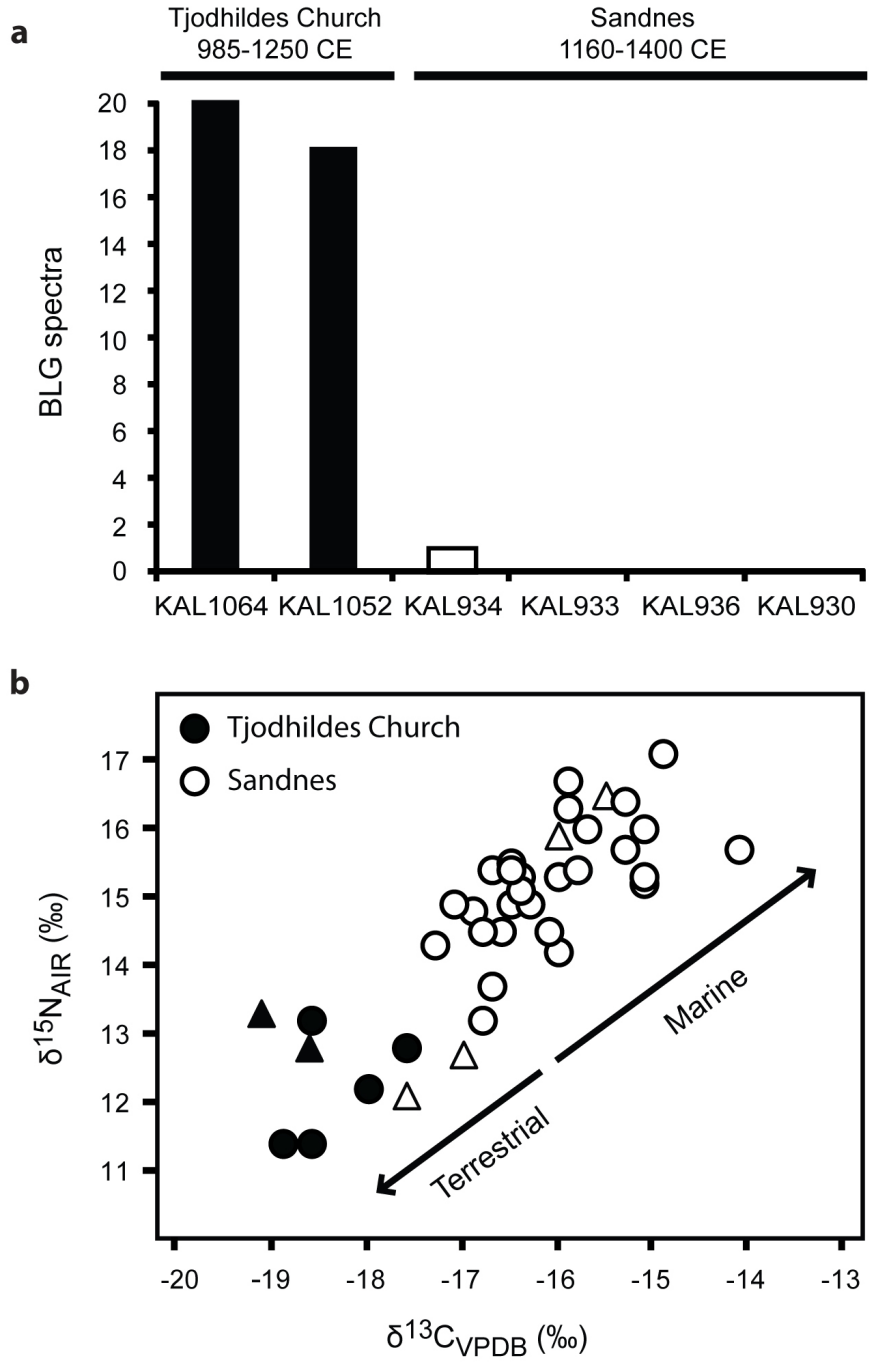
BLG pattern in dental calculus is consistent with bone collagen stable isotope evidence of a decline of the dairy economy in Norse Greenland with the onset of the Little Ice Age (ca.1250 CE). (a) Total spectra matching BLG peptides recovered from dental calculus samples from the earlier Tjodhildes Church at Ø29a Brattahlið in the Eastern Settlement (individuals KAL1064 and KAL1052) and from the later V51 Sandnes site in the Western Settlement (individuals KAL934, KAL933, KAL936, and KAL930). (b) Bone collagen carbon and nitrogen stable isotope values measured from burials at Tjodhildes Church (black) and Sandnes (white)^22^,^23^, showing a major dietary shift toward marine resources at the later Sandnes site. Isotopic values for individuals also analyzed for dental calculus BLG peptides are represented by triangles, and from left to right on the x-axis are: KAL1064, KAL1052, KAL934, KAL933, KAL936, and KAL930. Isotopic data for KAL1064 and KAL1052 were measured in this study.

Recent isotopic and faunal evidence suggests that Greenland Norse settlements shifted from an economy initially based on dairy to one increasingly reliant on marine mammals after the onset of the Little Ice Age ca. 1250 CE^23^. To test this hypothesis, we analyzed dental calculus from individuals buried at Tjodhildes Church, an early cemetery (ca. 985–1250 CE) at the Eastern settlement landnám site of Brattahlið (Qassiarsuk) established by Erik the Red, the founder of the Norse Greenland colonies^23^, and at Sandnes (Kilaarsarfik), a high-status farm and church in the Western Settlement that continued to be in use until the abandonment of the settlement in the 15^th^ century CE. The individuals analyzed from Tjodhildes Church (n = 2) exhibited strong evidence of dairy consumption (Fig. 3), with a total of 38 spectra matching BLG peptides (12 unique peptides including one *Bos*-specific sequence). Because these two individuals had not been previously analyzed isotopically, we then performed carbon and nitrogen stable isotope analysis on bone collagen extracted from these individuals and confirmed that their isotopic values are consistent with a terrestrial diet (Figure 3). Analysis of the Sandnes individuals (n = 4) revealed that only one individual showed even weak evidence of milk consumption, as evidenced by a single spectrum (Fig. 3). This individual was previously determined to have consumed a primarily terrestrial diet on the basis of isotopic evidence^23^. The remaining BLG-negative individuals exhibited bone stable isotope values consistent with increasing marine resource consumption (Figure 3).

## Discussion

Our results show that the milk protein BLG preserves in archaeological dental calculus and can be identified in specimens dating back to at least the Bronze Age (ca. 3000 BCE) in Europe and northern Southwest Asia. Moreover, we demonstrate that BLG is a species-specific milk biomarker that allows cattle, sheep, and goat dairy product consumption to be distinguished. As expected, no BLG was detected in specimens from Central West Africa, where dairy consumption was historically very low or absent and current LP frequencies are very low.

In addition to establishing BLG as a biomarker of milk consumption in the archaeological record, the broad survey of dental calculus BLG conducted in this study reveals previously uncharacterized temporal and geographical complexities in dairy consumption. In Central Europe, for example, it is intriguing given the high prevalence of LP in both modern day (Fig. 1) and medieval^27^ German populations that no BLG peptides were detected in Bronze Age or medieval German samples (0/9 individuals) (Table 1). This stands in contrast to the northern Italian and Hungarian samples where BLG peptides were detected at a relatively high frequency (5/11 individuals) (Table 1; Supplementary Table S2). These geographic patterns may reflect different dairy consumption levels or differential usage of high (e.g., soured milk, whey, ricotta, kefir) and low (e.g., cheese, butter) BLG dairy products. Using this approach, it now becomes possible to explore specific cultural, social, and environmental factors influencing past dairy economies at both a population and an individual level.

To explore a specific dairy economy in greater detail, we applied this approach to Norse Greenland to examine the hypothesis that this population underwent a dramatic dietary shift in response to environmental change during the Little Ice Age^23^. The medieval Greenland Norse economy was primarily based on animal husbandry and especially dairying. The short growing season and cold climate of Greenland precluded the successful establishment of agriculture but was sufficient for ruminant pastoralism. Like other Scandinavian populations, the medieval Norse utilized nearly all by-products of dairy processing, including BLG-rich whey^28^,^29^. The use of sour whey (*súrr*) to pickle meats is described in the Norse Icelandic sagas^30^, and Scandinavian whey “cheese” (e.g., *mysost, gjetost*, and *brunost*), an evaporated whey concentrate with an exceptionally high lactose content (30–55%), was also likely consumed, but unfortunately the sagas lack detail on specific “cheese” (*ost*) types.

Our dental calculus BLG results confirm that dairy products were consumed by individuals buried in the early Norse cemetery at Tjodhildes Church (ca. 985–1250 CE), and bone collagen stable isotopic values from the same individuals are consistent with a terrestrial diet. By contrast, BLG was very low or absent in the dental calculus of individuals buried at the Sandnam site, which was occupied until ca. 1430 CE, and bone stable isotope values from these individuals indicate a dietary shift toward marine resources.

Diminished access to dairy products and a collapse of dairy herds would have had a strongly negative effect on the Norse economy, removing not only a major source of storable nutrition but also impeding the ability to preserve other perishable foods, such as meats, thereby exacerbating food instability especially during the winter months. Our findings support the hypothesis that climate change and the consequent decline of dairy herds contributed to the decline and ultimate abandonment of the Norse Greenland colonies in the 15^th^ century CE.

In this study, we identify the protein β-lactoglobulin in archaeological dental calculus and demonstrate that it is a species-specific milk biomarker and an indicator of dairy consumption in the archaeological record. Nearly ubiquitous and obtained directly from the oral cavity of individuals, dental calculus provides a novel approach to detecting patterns of milk consumption and the dietary variables driving recent natural selection in humans.

## Methods

### Interpolated contour map of present day lactase persistence

Lactase persistence frequency data was estimated from allele frequency data assuming dominant inheritance and was taken, where possible, from full sequencing of the lactase enhancer to include all 5 published known functional LP variants^31^ with additional data taken from genotyping of the individual SNPs, where informative, as recorded in the GLAD database http://www.ucl.ac.uk/mace-lab/resources/glad^24^ but revised and updated to include more recent publications through March 2014^25^. Latitude and longitude of the data points were taken as near as possible to the collection sites where these were known. Where country alone was known these were estimated using major cities. The contour map was constructed in ‘R’ (v.3.1.0, 2014-04-10, “Spring Dance”) using the *spatstat* package^32^ and included weighting for sample size. Interpolation smoothing was conducted at the lowest non-overflowing bandwidth (value of sigma) allowable from the heterogeneous data available. Interpolation may be inaccurate where there are few data points, and it should be noted that neighboring populations with different ancestry and life-style, in Africa particularly, sometimes have very different allele frequencies.

### Samples and MS/MS analysis

Dental samples (n = 98) were obtained from diverse historic human populations in Eurasia, Africa, and Greenland dating from the Bronze Age to the present (Supplementary Table S1). Dental calculus was removed using a dental scaler and stored in sterile 2.0 mL tubes until further analysis. Tryptic peptides were extracted from decalcified dental calculus using a filter-aided sample preparation (FASP) protocol modified for degraded samples^33^ according to previously published protocols^18^. The extracted peptides were then analyzed using shotgun protein tandem mass spectrometry (MS/MS) to detect the presence of β-lactoglobulin. MS/MS analysis of samples were performed at three independent laboratories in Switzerland, the UK, and Denmark:

#### Functional Genomics Center Zurich at the University of Zurich and Swiss Federal Institute of Technology

Samples from Greenland and Germany (Z1, Z2, Z27, Z46, and Z28) were analyzed by tandem mass spectrometry at the Functional Genomics Centre Zürich (FGCZ) using an LTQ-Orbitrap VELOS mass spectrometer (Thermo Fischer Scientific, Bremen, Germany) coupled to an Eksigent-NanoLC-Ultra 1D plus HPLC system (Eksigent Technologies, Dublin (CA), USA). Solvent composition at the two channels was 0.2% formic acid, 1% acetonitrile for channel A and 0.2% formic acid, 100% acetonitrile for channel B. Peptides were loaded on a self-made tip column (75 μm × 80 mm) packed with reverse phase C18 material (AQ, 3 μm 200 Å, Bischoff GmbH, Leonberg, Germany) and eluted with a flow rate of 250 nl per min by a gradient from 0.8% to 4.8% of B in 2 min, 35% B at 57 min, 48% B at 60 min, 97% at 65 min. Full-scan MS spectra (300−1700 m/z) were acquired in the Orbitrap with a resolution of 30000 at 400 m/z after accumulation to a target value of 1,000,000. Higher energy collision induced dissociation (HCD) MS/MS spectra were recorded in data dependent manner in the Orbitrap with a resolution of 7500 at 400 m/z after accumulation to a target value of 100, 000. Precursors were isolated from the ten most intense signals above a threshold of 500 arbitrary units with an isolation window of 2 Da. Three collision energy steps were applied with a step width of 15.0% around a normalized collision energy of 40% and an activation time of 0.1 ms. Charge state screening was enabled excluding non-charge state assigned and singly charged ions from MS/MS experiments. Precursor masses already selected for MS/MS were excluded for further selection for 45 s with an exclusion window of 20 ppm. The size of the exclusion list was set to a maximum of 500 entries.

#### Proteomics Discovery Institute at the University of Oxford

Samples from Britain, Germany (Y47, Y48, and Y49), St Helena, and Italy (ODN19-1, ODN98-1, ODN207-1, ODN271-1, ODN361-1, ODN424-1, ODN458-1, SCR227-1, SCR250-1, SCR264-1, SCR323-1, SCR832-1, SCR5082-1, SCR5042-1, SCR5070-1) were analyzed by tandem mass spectrometry at the Central Proteomics Facility, Target Discovery Institute, Oxford on Q-Exactive and Orbitrap Elite tandem mass spectrometers.

Q-Exactive analysis was performed after UPLC separation on an EASY-Spray column (50 cm × 75 μm ID, PepMap RSLC C18, 2 μm) connected to a Dionex Ultimate 3000 nUPLC (all Thermo Scientific) using a gradient of 2–40% Acetonitrile in 0.1% Formic Acid and a flow rate of 250 nl/min @40°C. MS spectra were acquired at a resolution of 70000 @200 m/z using an ion target of 3E6 between 380 and 1800 m/z. MS/MS spectra of up to f15 precursor masses at a signal threshold of 1E5 counts and a dynamic exclusion for 7 seconds were acquired at a resolution of 17500 using an ion target of 1E5 and a maximal injection time of 50 ms. Precursor masses were isolated with an isolation window of 1.6 Da and fragmented with 28% normalized collision energy.

Orbitrap Elite analysis was performed under similar LC conditions as above using a nAcquity UPLC (1.7 um BEH130 C18, 75 um × 250 mm). MS spectra were acquired at a resolution of 120000 @ 400m/z using an ion target of 5E5 between 300 and 1800 m/z. MS/MS spectra of up to 200 precursor masses at a signal threshold of 1000 counts and a dynamic exclusion for 15 seconds were acquired in the linear ion trap using rapid scan and an ion target of 5E4. Precursor masses were isolated with an isolation window of 1.5 Da and fragmented with 35% normalized collision energy.

#### Novo Nordisk Foundation Center for Protein Research at the University of Copenhagen

Samples from Norway, Denmark, Hungary, Germany (RISE 472 and RISE 473), Italy (RISE 466 and RISE 467), Armenia, and Russia were analyzed by tandem mass spectrometry at the Novo Nordisk Foundation Center for Protein Research at the University of Copenhagen, Denmark using a Q-Exactive mass spectrometer. The LC-MS system consisted of an EASY-nLC™system (Thermo Scientific, Odense, Denmark) connected to the Q-Exactive (Thermo Scientific, Bremen, Germany) through a nano electrospray ion source. 5 uL of each peptide sample was auto-sampled onto and directly separated in a 15 cm analytical column (75 μm inner diameter) in-house packed with 3 μm C18 beads (Reprosil-AQ Pur, Dr. Maisch) with a 130 minute linear gradient from 5% to 25% acetonitrile followed by a steeper linear 20 minute gradient from 25% to 40% acetonitrile. Throughout the gradients a fixed concentration of 0.5% acetic acid and a flow rate of 250 nL/min were set. A final washout and column re-equilibration added an additional 20 minutes to each acquisition. The effluent from the HPLC was directly electrosprayed into the mass spectrometer by applying 2.0 kV through a platinum-based liquid-junction. The Q-Exactive was operated in data-dependent mode to automatically switch between full scan MS and MS/MS acquisition. Software control was Tune version 2.2–1646 and Excalibur version 2.2.42, and the settings were adjusted for ‘sensitive’ acquisition. Briefly, each full scan MS was followed by up to 10 MS/MS events. The isolation window was set at 2.5 Th and a dynamic exclusion of 90 seconds was used to avoid repeated sequencing. Only precursor charge states above 1 and below 6 were considered for fragmentation. A minimum intensity threshold for triggering fragment MS/MS was set at 1e5. Full scan MS were recorded at resolution of 70,000 at m/z 200 in a mass range of 300–1750 m/z with a target value of 1e6 and a maximum injection time of 30 ms. Fragment MS/MS were recorded with a fixed ion injection time set to 108 ms through a target value set to 2e5 and recorded at a resolution of 35,000 with a fixed first mass set to 100 m/z. Normalized collision energy was 25%.

### Data analysis

Raw MS/MS spectra were converted to searchable Mascot generic format using Proteowizard version 3.0.4743 using the 200 most intense peaks in each MS/MS spectrum. MS/MS ion database searching was performed on Mascot (Matrix Science™, version 2.4.01), against all available sequences in UniProt and the Human Oral Microbiome Database (HOMD)^34^. Searches were performed against a decoy database to generate false discovery rates. Peptide tolerance was 10 ppm, and with a semi-tryptic search with up to two missed cleavages. MS/MS ion tolerance was set to 0.07 Da. Based on previous observations of ancient proteome degradation^35^, we set post-translational modifications were as carbamidomethylation (fixed modification) and acetyl (protein N-term), deamidated (NQ), glutamine to pyroglutamate, methionine oxidation and hydroxylation of proline (variable modifications). Mascot search results were filtered using an ion score cut-off of 25 and significance threshold of *p* < 0.05. BLAST was used to verify matches to β-lactoglobulin, and taxonomic assignment is reported based on the consensus peptide assignments for each individual. The three-dimensional structure of bovine β-lactoglobulin protein, rendered from PDB 3NPO using Visual Molecular Dynamics software^33^ VMD v.1.9.1, http://www.ks.uiuc.edu/Research/vmd/current/.

### Contamination exclusion

It is important to monitor and test for contamination because bovine proteins are used in some proteomics laboratories as instrument standards (e.g., bovine fetuin), among other purposes. In order to exclude such contaminants as the source of the BLG peptides in dental calculus, negative extraction controls, bovine fetuin protein standards, and isopropanol wash steps were analyzed with the experimental samples in parallel. No BLG peptides were observed in any non-template negative extraction controls (n = 12), bovine fetuin standards (n = 21), or isopropanol wash steps (n = 9).

### BLG protein modeling

The three-dimensional structure of bovine β-lactoglobulin protein was rendered from the Research Collaboratory for Structural Bioinformatics Protein Data Bank (RCSB PDB) accession 3NPO (unliganded, DOI:10.2210/pdb3npo/pdb) using VMD v.1.9.1^36^. The mapped locations of all BLG peptide sequences identified by tandem mass spectrometry within archaeological dental calculus were then visualized in red, while unmapped regions were visualized in white.

### Bone collagen stable isotope analysis

Bone collagen from Tjodhilde's Church individuals KAL1052 and KAL1064 was prepared for stable isotope δ^13^C and δ^15^N analysis as previously described^37^. Duplicate collagen specimens (1 mg) were measured using a Sercon 20–22 Isotope Ratio Mass Spectrometer coupled to a Sercon GSL Elemental Analyser in the Department of Archaeology at the University of York. The results for KAL1052 are as follows: δ^13^C, −18.7352, −18.5608; δ^15^N, 12.8049, 12.8799; C/N, 3.31. The results for KAL1064 are as follows: δ^13^C, −19.1342, −19.1654; δ^15^N, 13.1990, 13.3332; C/N, 3.51. Carbon isotopic values are reported relative to Pee Dee Belemnite (PDB); nitrogen isotopic values are reported relative to AIR. Mean bone collagen δ^13^C and δ^15^N for each sample are presented in Figure 3. Stable isotopic data for the Sandnes samples and the remaining Tjodhilde's Church samples were obtained from the literature^22^,^23^.

## Author Contributions

C.W., C.S., J.H., M.J.C., E.C. and M.T.P.G. designed the research. C.W., C.T., J.H., C.S., R.F., S.F., E.C., A.L., A.F., R.J.C., J.O., S.C. and M.M. performed experiments. C.W., J.H., S.F., C.S., M.J.C., J.A., J.G., A.L., N.M. and D.S. analyzed data. M.J.C., C.S., J.H., M.T.P.G., E.C., A.B., F.R., D.S., A.C., O.E.C. and N.L. provided materials and resources. C.W., J.H. and M.J.C. wrote the paper, with input from the other co-authors.

## Additional Information

**Accession codes**: Data are available via ProteomeXchange with identifiers PXD001357, PXD001359, PXD001360, PXD001361, and PXD001362.

## Supplementary Material

Supplementary Information Supplementary Figure S1

Supplementary Information Supplementary Table S1

Supplementary Information Supplementary Table S2

## Figures and Tables

**Table 1:** Proteomic results of archaeological dental calculus samples analyzed in this study

			BLG
Region	Dates	N	Individuals (spectra)
*Northern Europe*			
Britain	ca. 800 BCE to 1895 CE	33	11 (128)
Denmark	ca. 3000–1500 BCE	2	0 (0)
Norway	1100–1700 CE	5	* (43)
*Central Europe*			
Germany	ca. 3000 BCE to 1200 CE	9	0 (0)
Hungary	ca. 3000–1500 BCE	2	1 (38)
Italy	ca. 2700 BCE to 200 CE	17	4 (11)
*Northern Southwest Asia*			
Armenia	ca. 2000–700 BCE	4	1 (2)
Russia	ca. 3000–1500 BCE	2	1 (8)
*Central West Africa*			
St. Helena	ca. 1840–1872 CE	18	0 (0)
*Greenland (Norse)*			
Eastern Settlement	ca. 890–1230 CE	2	2 (38)
Western Settlement	ca. 1290–1430 CE	4	1 (1)

*Notes*:

*Extracted proteins from five individuals were pooled for analysis.
