# Supplementary material for: Direct evidence of milk consumption from ancient human dental calculus
Source: Sci Rep. 2014 Nov 27;4:7104. doi: 10.1038/srep07104 (PMC4245811; doi:10.1038/srep07104)
Supplement: Supplementary Information — Supplementary Figure S1 [file srep07104-s1.pdf]

Supplementary Information

Direct evidence of milk consumption from ancient human dental calculus

Warinner C*, Hendy J*, Speller C, Cappellini E, Fischer R, Trachsel C, Arneborg J, Lynnerup N, Craig OE, Swallow DM, Fotakis A, Christensen RJ, Olsen JV, Liebert A, Montalva N, Fiddyment S, Charlton S, Mackie M, Canci A, Bouwman A, Rühli F, Gilbert MTP, Collins MJ

*These authors contributed equally to this work.

Correspondence and requests for materials should be made to Christina Warinner, twarinner@gmail.com or to Matthew Collins, matthew.collins@york.ac.uk

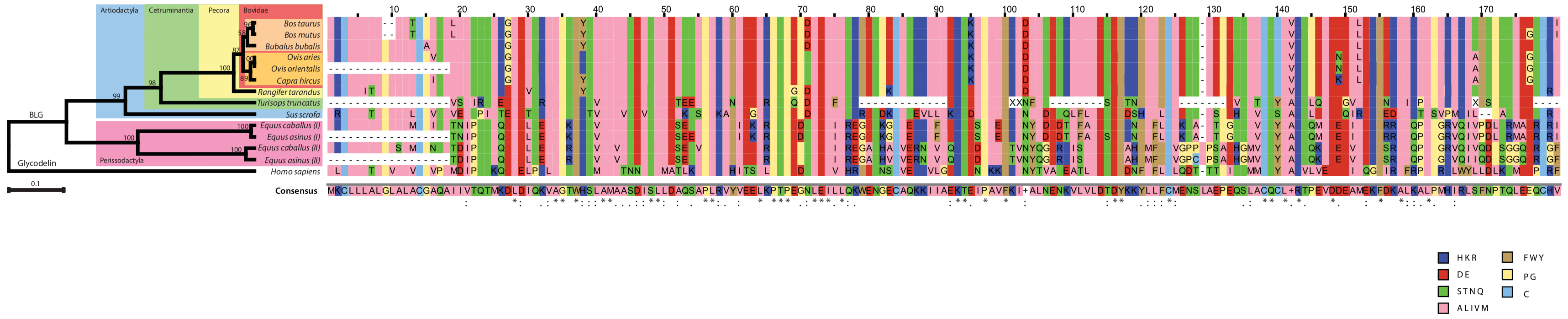

Supplementary Figure 1 | UPGMA phylogram and sequence alignment of BLG in traditional dairy livestock and other animals. Reference sequence citations and additional information are provided in Supplementary Table 1. In the phylogram, relevant taxonomic groups are highlighted: Bovinae subfamily (pink), Caprinae subfamily (orange), Bovidae family (red), Pecora infraorder (yellow), Cetruminantia suborder (green), Artiodactyla order (blue), Perissodactyla order (purple). Camelids and humans do not produce BLG; glycodelin, the human protein most closely related to BLG, is provided for comparison. The consensus sequence is presented below the protein alignment, and annotated as follows: single, fully conserved residue (*); conservation of strongly similar properties, >0.05 in Gonnet PAM 250 matrix (:); conservation of weakly similar properties, >0.05 in Gonnet PAM 250 matrix (.). Specific residues differing from the consensus sequence are indicated in the alignment, and gaps are signified by dashes (-). Residue similarity is indicated using a modified CINEMA amino acid color scheme: polar positive HKR (dark blue), polar negative DE (red), polar neutral STNQ (green), non-polar aliphatic ALIVM (pink), non-polar aromatic FWY (brown), non-polar aromatic PG (yellow), non-polar aromatic C (light blue). All BLG peptides identified within human dental calculus in this study (Supplementary Table 1) are specific to the Pecora infraorder of Artiodactyla and include diagnostic polymorphisms for cattle (*Bos* sp.), sheep (*Ovis* sp.), and goats (*Capra* sp.).
